# The effect of combining green iron nanoparticles and algae on the sustainability of broiler production under heat stress conditions

**DOI:** 10.3389/fvets.2024.1359213

**Published:** 2024-02-21

**Authors:** Yousri A. R. Almeldin, Amira E. Eldlebshany, Enass Abd Elkhalek, Ahmed A. A. Abdel-Wareth, Jayant Lohakare

**Affiliations:** ^1^Poultry Science Department, Faculty of Agriculture, Alexandria University, Alexandria, Egypt; ^2^Poultry Center, Cooperative Agricultural Research Center, Prairie View A and M University, Prairie View, TX, United States; ^3^Department of Animal and Poultry Production, Faculty of Agriculture, South Valley University, Qena, Egypt

**Keywords:** algae, Ammonia, broilers, growth performance, Iron Fe nanoparticles, meat quality

## Abstract

**Background:**

Natural feed additives in broiler feed contribute to the overall health, productivity, and economic viability of broiler chickens while meeting consumer demands and preferences for natural products. The purpose of this research was to determine the effect of green iron nanoparticles (Nano-Fe) and *Halimeda opuntia* supplementation in broiler diets on performance, ammonia excretion in excreta, Fe retention in tissues and serum, carcass criteria, and meat quality under hot environmental conditions.

**Methods:**

A total of 256 one-day-old male Ross 308 broiler chicks were randomly assigned to one of four feeding treatments for 42 days. Each treatment had eight replications, with eight chicks per replicate. The treatments were Negative control (CON), positive control (POS) supplemented with 1 g/kg *Halimeda opuntia* as a carrier, POS + 20 mg/kg Nano-Fe (NFH1), POS + 40 mg/kg Nano-Fe (NFH2).

**Results:**

When compared to CON and POS, dietary Nano-Fe up to 40 mg/kg enhanced (*p* < 0.001) growth performance in terms of body weight (BW), body weight gain (BWG), and feed conversion ratio (FCR). Nano-Fe had the highest BWG and the most efficient FCR (linear, *p* < 0.01, and quadratic, *p* < 0.01) compared to POS. Without affecting internal organs, the addition of Nano-Fe and POS enhanced dressing and reduced (*p* < 0.001) abdominal fat compared to control (CON). Notably, the water-holding capacity of breast and leg meat was higher (*p* < 0.001), and cooking loss was lower in broilers given Nano-Fe and POS diets against CON. In comparison to POS, the ammonia content in excreta dropped linearly as green Nano-Fe levels increased. When compared to CON, increasing levels of Nano-Fe levels boosted Fe content in the breast, leg, liver, and serum. The birds fed on POS showed better performance than the birds fed on CON.

**Conclusion:**

Green Nano-Fe up to 40 mg/kg fed to broiler diets using 1 g/kg *Halimeda opuntia* as a carrier or in single can be utilized as an efficient feed supplement for increasing broiler performance, Fe retentions, carcass characteristics, meat quality, and reducing ammonia excretions, under hot conditions.

## 1 Introduction

Food safety is a significant concern in hot climatic regions of the world, particularly for chicken meat, eggs, and other products consumed by humans. Heat stress frequently reduces feed intake, impairs growth rates, contributes to increased mortality rates, alters meat quality, causes economic losses, and affects physiological health, immunity, and respiratory distress in broiler chickens (1). The harmful effects of heat stress in chickens have been lessened by the use of several mitigating techniques. The rapidly developing field of nanotechnology has many potential uses in poultry feeding. Nanotechnology uses materials with new, distinctive properties between 1 and 100 nm in size (2). The physical and chemical characteristics of nanoparticles are different from those of their original, equivalent material, which can increase their bioavailability (3, 4). The nanoparticle’s lower antagonism in the gut results in better absorption, less excretion into the environment, and higher feed efficiency (5).

Iron (Fe) is a necessary mineral that is frequently added to the diet of broilers. It plays a crucial position in a variety of enzymes and proteins that govern cell development and differentiation, transport oxygen, and preserve health (6, 7). It contributes significantly to the tricarboxylic acid cycle by supporting enzymes, which facilitates the removal of harmful metabolites by catalases and peroxidases with iron (8). Heat stress lowers the amounts of Fe in serum and tissue (9). A reduction in Fe causes the immune and antioxidant systems to malfunction, which is harmful to birds’ health (10). This element is abundant in nature and is found in all components used in commercial poultry diets (11). Absorption and transport of dietary Fe across the intestinal mucosa occur in mechanisms that are strongly reliant on Fe status (12). Furthermore, Fe is mostly bound to phytate in cereals and oilseeds (13), which reduces its availability in poultry diets when phytase is not added (14). However, in animals, it is mostly present in myoglobin, cytochromes, hemoglobin (60–70%), ferritin, and hemosiderin (20–30%), as well as other Fe-containing enzymes (10%) (15). Because heme Fe has a preferred absorption pathway over inorganic Fe, animal byproducts containing muscle tissue and blood have higher Fe availability for poultry (16). Green nanotechnology refers to the use of environmentally friendly processes and materials in the synthesis of nanoparticles. This approach often involves using natural sources, such as plant extracts or microorganisms, to reduce and stabilize nanoparticles.

Green Nano-iron (Nano-Fe) formulations may address challenges related to the bioavailability of iron in conventional feed resources and mineral salts. The use of green nanotechnology is driven by the desire to minimize the environmental impact and potential toxicity associated with traditional nanoparticle synthesis methods (2). The use of green Nano-Fe in poultry nutrition offers several potential benefits, combining the advantages of nanotechnology with environmentally friendly and sustainable practices. Green Nano-Fe particles have more surface area than typical iron sources. This increased surface area can improve iron bioavailability, allowing for improved nutrient absorption in poultry’s digestive system, which is crucial for broiler health and growth (5, 17, 18). Nanoparticles of Fe have been of interest in various fields, including agriculture and poultry production, due to their potential applications in areas such as nutrient delivery, disease treatment, and environmental remediation (17). Nano-Fe supplementation increased body weight in broiler meals without altering the composition of the liver, thigh, or breast (18).

*Halimeda opuntia*, commonly known as sea cactus, is a type of green algae that is found in marine environments. In addition, algae can be grown as ingredients and dietary supplements for poultry feed (19). According to Martins et al. (20), algae have a unique composition consisting of carbohydrates, proteins, lipids, vitamins, minerals, and bioactive substances including carotenoids. Microalgae are recommended as feed additives due to their high levels of macro- and micro-elements and ability to improve the growth performance, feed efficiency, and meat quality of broilers (21), which is primarily due to properties of polysaccharides that can increase the health and productivity of chickens.

However, there is no data on the effect of graded inclusion levels of green Nano-Fe and algae on broiler performance and meat quality under hot environmental conditions. We investigated the mechanism of the effects of green Nano-Fe supplementation on broiler productive performance under hot environmental conditions. We wanted to evaluate the effects of varied inclusion levels of green Nano-Fe in broiler diets using 1 g/kg algae as a carrier on growth performance, ammonia emission in excreta, Fe retention, carcass criteria, and meat quality under heat stress.

## 2 Materials and methods

### 2.1 Dietary treatments and experimental design

The animal study protocol was approved by the Institutional Animal Care and Use Committee of the University of Alexandria, Egypt (AU08220810298). A total of 256 one-day-old male Ross 308 broiler chicks were randomly assigned to one of four feeding treatments until they reached 42 days old. Each treatment had eight replications, with eight chicks per replicate. Negative control (CON), positive control (POS) supplemented with 1 g/kg microalgae as a carrier, POS + 20 mg/kg Nano-Fe (NFH1), and POS + 40 mg/kg Nano-Fe (NFH2) were the treatments. The 42-day experiment was divided into two phases (0 to 21 days for the starter and 21 to 42 days for the grower). The experimental diets used in the present study contained around 20 mg and 40 mg of green Nano-Fe/kg, which is below the minimum recommended level of 85 mg Fe/kg (11). Furthermore, the levels of Nano-Fe were selected based on previous studies suggesting that chicken diets containing varying amounts of Fe (from 10 to 60 mg/kg in non-supplemented diets to about 160 mg in diets supplemented with 140 mg Fe-Gly or 100 mg Nano-Fe) (22, 23). The diets were formulated to meet Ross 308 broiler recommendations (Table 1). Chicks had full access to feed and water during the experimental period. The experiment was conducted at the Poultry Center, Faculty of Agriculture, South Valley University. The cage measurements for the chickens were 120 × 70 × 50 cm in length, breadth, and height, respectively. There were four nipple drinkers and hanging linear feeders in each pen. As the birds grew, so did the height of the nipple line. A 23-h continuous light scheme was implemented from the first day to 42 days of age. The ambient temperature was gradually reduced from 34.5°C (45 RH%) for days 1 to 21 to 28.5°C, 40 RH%, and 29.9 temperature-humidity index (THI) from 22 to 42 days of age.

**Table 1:** The chemical composition of the basal diet (as-fed basis).

Ingredients, g/kg	Starter diet	Grower diet
Corn	276	300
Sorghum	276	300
Soybean (44% CP)	285	250
Corn gluten (60% CP)	95.0	60.0
Vit and Min. Premix^a^	3.00	3.00
Sunflower Oil	30.0	55.2
Dicalcium phosphate	20.0	18.0
Limestone	10.0	10.00
Salt	3.80	3.80
DL-methionine	0.40	---
L-lysine HCl	1.00	---
Total	1,000	1,000
Analyzed chemical composition, g/kg		
Dry matter	925	924
Crude protein	233	216
Ether extract	53.7	57.5
Crude fiber	25.8	37.8
Ash	67.4	61.8
Ca	13.22	12.84
P	7.05	7.21
Fe	0.024	0.026
GE, MJ/kg (Calculated)	18.55	19.18

a Supplied per kg diet: biotin (50 mg), pantothenic acid (10,000 mg), folic acid (1,000 mg), nicotinic acid (30,000 mg), vitamin A (1900 IU), K3 (1,000 mg), B1 (1,000 mg), B2 (5,000 mg), B6 (1,500 mg), and B12 (0.046 mg) in addition to D3 (1,300 IU), E (10,000 mg), and BHT (10,000 mg) and includes 60 mg of Mn, 50 mg of Zn, 0.1 mg of Se, 4 mg of Cu, 3 mg of I, and 0.1 mg of Co.

THI = db°C- [(0.31–0.31RH; db°C-14.4)], where db is the dry bulb temperature in degrees Celsius and RH is the relative humidity percentage/100. The calculated THI values were then classed as follows: 27.8 indicates no heat stress, 27.8–28.9 indicates moderate heat stress, 28.9–30.0 indicates severe heat stress, and > 30.0 indicates extremely severe heat stress (1).

### 2.2 Green synthesis of Fe nanoparticles

**Figure 1:**
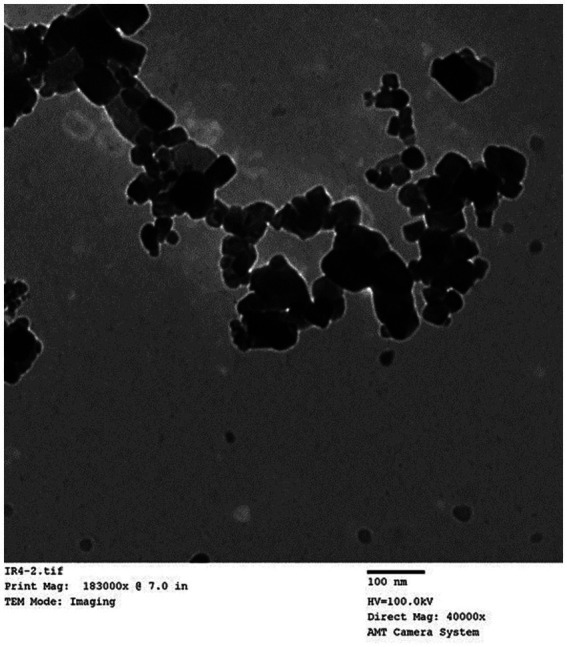
Transmission electron micrographs (TEM) of Nano-Fe.

In accordance with the maceration technique outlined by Khalil et al. (24), green Fe oxide nanoparticles were produced from the leaf extract of *Ocimum basilicum*. To sum up, a hot-plate magnetic stirrer was used to heat 30 g of plant powder and 200 mL of distilled water to 80°C for 1 h. Solid residues were eliminated from the final solution by filtering it three times with the Whatman No. 1 filter paper. The filtered solution with a pH of 5.7 was heated for 2 h at 85°C and then 100 mL of Fe (III) chloride (6 g) was added as a precursor salt. The solution went from being brownish to violet in hue, and its pH was recorded. After allowing the mixture to reach room temperature, decantation was used to extract the Fe oxide nanoparticles. After three cycles of distilled water washing, the Fe oxide was allowed to dry at room temperature. These particles were characterized by transmission electron microscope (TEM; Figure 1).

### 2.3 Algae preparations

The macroalga *Halimeda opuntia* was collected by hand-picking from the Red Sea in Hurghada, Egypt. Healthy algae samples were cleaned from epiphytes, extraneous matter, and necrotic were removed. Samples were washed thoroughly with sea water then sterile distilled water, air dried, cut into small pieces, and then ground in a tissue grinder to pass through a 1 mm screen [IKA A 10, Germany] until reached a fine powder shape. The ground *Halimeda opuntia* was kept until used to mix with the experimental diets. One g/kg of *Halimeda opuntia* macroalgae was added to broiler diets, and this dose was chosen in compliance with previous studies suggesting that the ideal levels of macroalgae in broiler diets should range from 0.5 and 3 g/kg (19–21).

### 2.4 Broiler productive performance

From the first day of the experiment to the last, the body weight (BW) was recorded for each pen once a week. Furthermore, on the day that the birds were weighed, feed residue was measured in order to calculate the amount of feed that each pen consumed in between weigh-ins. The feed conversion ratio (FCR) was calculated by dividing the weight of feed consumed by the body weight gain (BWG) of each pen. This yielded the feed per gain. A correction for bird mortalities was applied to the magnitude of production variables, such as feed consumption and body weight.

### 2.5 Ammonia analysis

The excreta was collected daily at 21 to 42 days of age per pen for the determination of ammonia excretion according to the method proposed by Miles et al. (25). 200 g of freshly collected excrement was added to a 1,000 mL jar. A rubber stopper sealed the upper portion of the jar; the rubber plug featured an exhaust pipe and an intake pipe that linked to a U-shaped bubble absorption tube. The U-shaped bubble absorption tube (which was shielded from light) received around 10 mL of 2% boric acid. The U-shaped absorption tube’s other end was linked to an inflating pump via a second buffering device, and the intake pipe was connected to a buffer device. In an acidic environment, the 2% boric acid absorption solution was used to repair the ammonia gas produced from chicken feces, creating a stable NH4+. By using the Kjeldahl nitrogen determination method, the nitrogen content in the absorption solution was ascertained. The nitrogen content was then translated into NH3 content in the unit mass of feces (fresh weight basis).

### 2.6 Carcass criteria and internal organs

At 42 days of age, 40 birds per treatment were selected at random (five birds per replicate pen), weighed, slaughtered according to the Halal method, and plucked. Weighing the remaining portion of the body after the head, neck, viscera, digestive tract, shanks, spleen, liver, heart, gizzard, and abdominal fat were removed allowed us to calculate the relative weight. The formula for calculating dressing percentage is dressed weight/live weight × 100. The percentage of abdominal fat, liver, heart, and empty gizzard were calculated based on live body weight.

### 2.7 Meat quality measurements

The water-holding capacity (WHC) and cooking loss were assessed from the left side of the breast muscle and the left leg in 40 birds per treatment which were randomly selected (5 birds per replicate). The low-speed centrifugation technique was utilized to quantify the WHC of breast muscles with minimal adjustments (26). 10 g of intact breast muscle was placed in a falcon tube with glass beads and centrifuged for 20 min at 10,000 g at 5°C. The precipitated meat was then removed immediately, dried with filter paper, and weighed once more. The WHC was calculated using the weight loss in muscle samples after centrifugation. Cooking loss was calculated, as previously stated (27). In summary, the muscle filets were placed separately in thin-walled thermotolerant polyethylene bags and cooked in a water bath until their core temperatures reached 70°C. Following that, they were refrigerated in crushed ice until they reached 5°C, and the cooking loss was calculated by reweighing them. Samples of the liver, breast, leg, and blood were taken and held at −20°C for the Fe chemical analysis.

### 2.8 Fe analysis

For each treatment, 40 were randomly selected and the birds’ wing veins were utilized to extract blood (5 birds were used for each replicate), which was then placed in vacutainer tubes to collect serum. After 40 birds were killed, samples of their breast, leg, liver, and serum were taken, and they were promptly frozen at −20°C to be subjected to a Fe content study. The Fe concentrations were measured using an atomic absorption spectrophotometer (Perkin Elmer Analyst 800 model, Shelton, CT, United States).

### 2.9 Statistical analysis

The General Linear Models (GLM) technique for statistical analysis and SAS 9.2 software was used to examine the data of a completely randomized trial (SAS Institute) (28). The only constant in the model was the dosage of the supplements. The birds served as the experimental units for ammonia, carcass criteria, meat quality, and Fe retention, while the pen served as the experimental unit for growth performance. One-way ANOVA was utilized to examine the data, and Duncan multiple range tests were employed to compare means. The graphs were created using GraphPad Prism software, version 9 (GraphPad Software, La Jolla, CA, United States), along with a normal distribution test (Anderson—Darling test for normality). The linear and quadratic impacts of increasing Nano-Fe supplementations were calculated using orthogonal polynomial contrasts, with only POS (0 mg/kg Nano-Fe) taken into consideration as a control, and CON was not included in this analysis. A significance value of *p* < 0.05 was used. *p* values less than 0.001 were expressed as ‘<0.001’ rather than the actual value.

## 3 Results

### 3.1 Productive performance

The effects of green Nano-Fe on the BW and BWG of broiler chickens during the starter phase (0 to 21 d) and grower phase (22 to 42 d) are shown in Table 2. When compared to CON, dietary Nano-Fe up to 40 mg/kg enhanced (*p* < 0.001) BW and BWG. Nano-Fe at 20 mg/kg and 40 mg/kg with 1 g/kg *Halimeda opuntia* as a carrier in broiler diets increased (*p* < 0.05) BW compared to CON and POS during 21 and 42 days of age. There was an increase (*p* < 0.05) in BWG in POS when compared with CON during 1–21, 22–42, and 1–42 days of age showing the positive effects of adding algae. Similarly, dietary treatments containing POS, Nano-Fe at 20 mg/kg, and Nano-Fe at 40 mg/kg increased BWG (*p* < 0.001) by 10.95, 10.30, and 14.50%, respectively, compared to control throughout the trial period (1–42 days). Considering the entire trial period, feed intake in the supplemented groups differed significantly, although the Nano-Fe at 40 mg/kg showed the highest feed intake compared to others (Table 3). The addition of Nano-Fe improved (*p* < 0.05) FCR when compared to CON and POS at 1–21, 22–42, and 1–42 days of age. The CON group performed the worst in terms of BW, BWG, and FCR when compared to the POS and Nano-Fe groups. POS, Nano-Fe at 20 mg/kg, and Nano-Fe at 40 mg/kg feeding treatments improved the FCR (*p* < 0.006) compared to the CON throughout the trial (1–42 days).

**Table 2:** Effects of green Nano-Fe and algae on body weight and body weight gain of broiler chickens.

Items	Body weight, g/bird			Body weight gain, g/bird		
	1 day	21 days	42 days	1–21 days	21–42 days	1–42 days
Treatments, mg/kg						
CON	42.50	709.5^d^	1939.5^c^	667.0^d^	1230.0^c^	1897.0^c^
POS	42.75	741.6^c^	2147.6^b^	698.8^c^	1406.0^b^	2104.8^b^
NFH1	42.38	772.0^b^	2156.2^b^	729.6^b^	1384.2^b^	2113.8^b^
NFH2	42.25	800.6^a^	2245.3^a^	758.3^a^	1445.7^a^	2203.3^a^
SEM	0.090	7.351	19.636	7.372	21.038	19.834
*p* value						
Treatment	0.240	<0.001	<0.001	<0.001	<0.001	<0.001
Linear	0.054	<0.001	0.002	<0.001	0.209	0.002
Quadratic	0.556	0.936	0.108	0.927	0.128	0.109

^a–d^Means with different superscript in a column are significantly different (*p* < 0.05). CON: negative control. POS: positive control (1 g/kg *Halimeda opuntia*). NFH1: 1 g/kg *Halimeda opuntia* with 20 mg/kg Nano-Fe. NFH2: 1 g/kg *Halimeda opuntia* with 40 mg/kg Nano-Fe. SEM: Standard error of the means (*n* = 8).

**Table 3:** Effects of green Nano-Fe and algae on feed intake and feed conversion ratio of broiler chickens.

Items	Feed intake, g/bird			Feed conversion ratio		
	1–21 days	21–42 days	1–42 days	1–21 days	21–42 days	1–42 days
Treatment, mg/kg						
CON	901.6^c^	2329.1^a^	3230.7^b^	1.352^a^	1.896^a^	1.703^a^
POS	947.5^b^	2340.6^a^	3288.2^b^	1.356^a^	1.667^b^	1.563^b^
NFH1	907.9^c^	2167.3^b^	3075.2^c^	1.244^b^	1.566^c^	1.455^c^
NFH2	986.4^a^	2469.3^a^	3455.8^a^	1.302^b^	1.708^b^	1.568^b^
SEM	7.759	30.909	34.626	0.012	0.031	0.021
*p* value						
Treatment	<0.001	0.002	0.001	0.001	<0.001	<0.001
Linear	<0.001	0.040	0.008	0.020	0.312	0.835
Quadratic	<0.001	<0.001	<0.001	<0.001	0.002	<0.001

^a–d^Means with different superscript in a column are significantly different (*p* < 0.05). CON: negative control. POS: positive control (1 g/kg *Halimeda opuntia*). NFH1: 1 g/kg *Halimeda opuntia* with 20 mg/kg Nano-Fe. NFH2: 1 g/kg *Halimeda opuntia* with 40 mg/kg Nano-Fe. SEM: Standard error of the means (*n* = 8).

### 3.2 Ammonia contents

**Figure 2:**
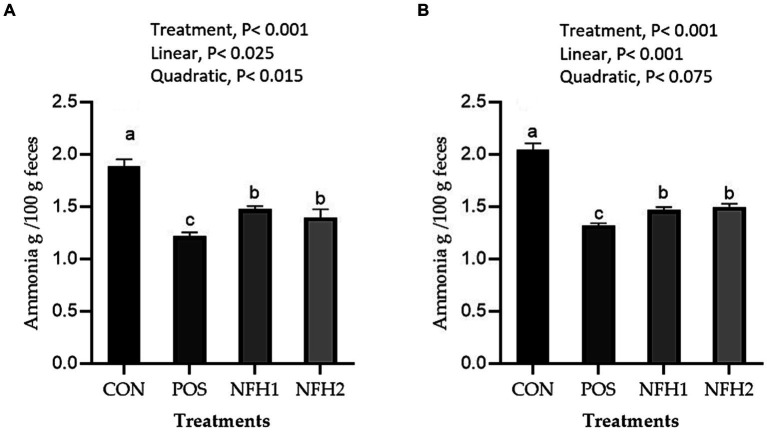
Effects of green Nano-Fe and algae on broiler chicken excreta ammonia contents at 21 **(A)** and 42 **(B)** days of age. Bars with different letters **(a–c)** are significantly different (*p* < 0.05). CON: negative control, POS: positive control (1 g/kg *Halimeda opuntia*), NFH1: 1 g/kg *Halimeda opuntia* with 20 mg/kg Nano-Fe, NFH2: 1 g/kg *Halimeda opuntia* with 40 mg/kg Nano-Fe, SEM: Standard error of the means (*n* = 40).

Figure 2 represents the effects of Nano-Fe supplementation on ammonia concentration at 21 and 42 days of age. Green Nano-Fe levels in broiler diets decreased (*p* < 0.001) excreta ammonia content when compared to CON and *Halimeda opuntia* alone at 21 and 42 days of age in heat stress. The POS group had the lowest (*p* < 0.01) level of excreta ammonia, followed by the Nano-Fe and CON groups. When POS was compared to green Nano-Fe levels in broiler diets at 21 and 42 days of age under heat stress, the excreta ammonia concentration reduced linearly (*p* < 0.001).

### 3.3 Carcass criteria

According to the carcass criteria (Table 4), broilers fed diets containing POS and POS including Nano-Fe at 20 mg/kg and 40 mg/kg showed increases (*p* < 0.05) in dressing percentage and decreases in abdominal fat at the end of the experiment compared to CON. Supplementation of POS and POS with Nano-Fe had no effect (*p* > 0.05) on the percentages of liver, heart, spleen, and gizzard of broilers compared to CON in heat stress.

**Table 4:** Effects of green Nano-Fe and algae on carcass characteristics at 42 days of age.

Items	Dressing%	Abdominal fat%	Liver%	Heart%	Gizzard%	Spleen %
Treatment, mg/kg						
CON	75.18^b^	0.833^a^	1.880	0.424	1.264	0.116
POS	78.74^a^	0.619^b^	1.935	0.464	1.344	0.117
NFH1	78.80^a^	0.547^b^	1.966	0.445	1.358	0.114
NFH2	78.82^a^	0.513^b^	1.885	0.455	1.305	0.115
SEM	0.326	0.035	0.038	0.010	0.030	0.005
*p* value						
Treatment	0.028	0.001	0.859	0.106	0.711	0.054
Linear	0.002	0.001	0.675	0.730	0.668	0.142
Quadratic	0.485	0.057	0.590	0.222	0.674	0.062

^a–d^Means with different superscript in a column are significantly different (*p* < 0.05). CON: negative control. POS: positive control (1 g/kg *Halimeda opuntia*). NFH1: 1 g/kg *Halimeda opuntia* with 20 mg/kg Nano-Fe. NFH2: 1 g/kg *Halimeda opuntia* with 40 mg/kg Nano-Fe. SEM: Standard error of the means (*n* = 8).

### 3.4 Physicochemical properties of meat

**Figure 3:**
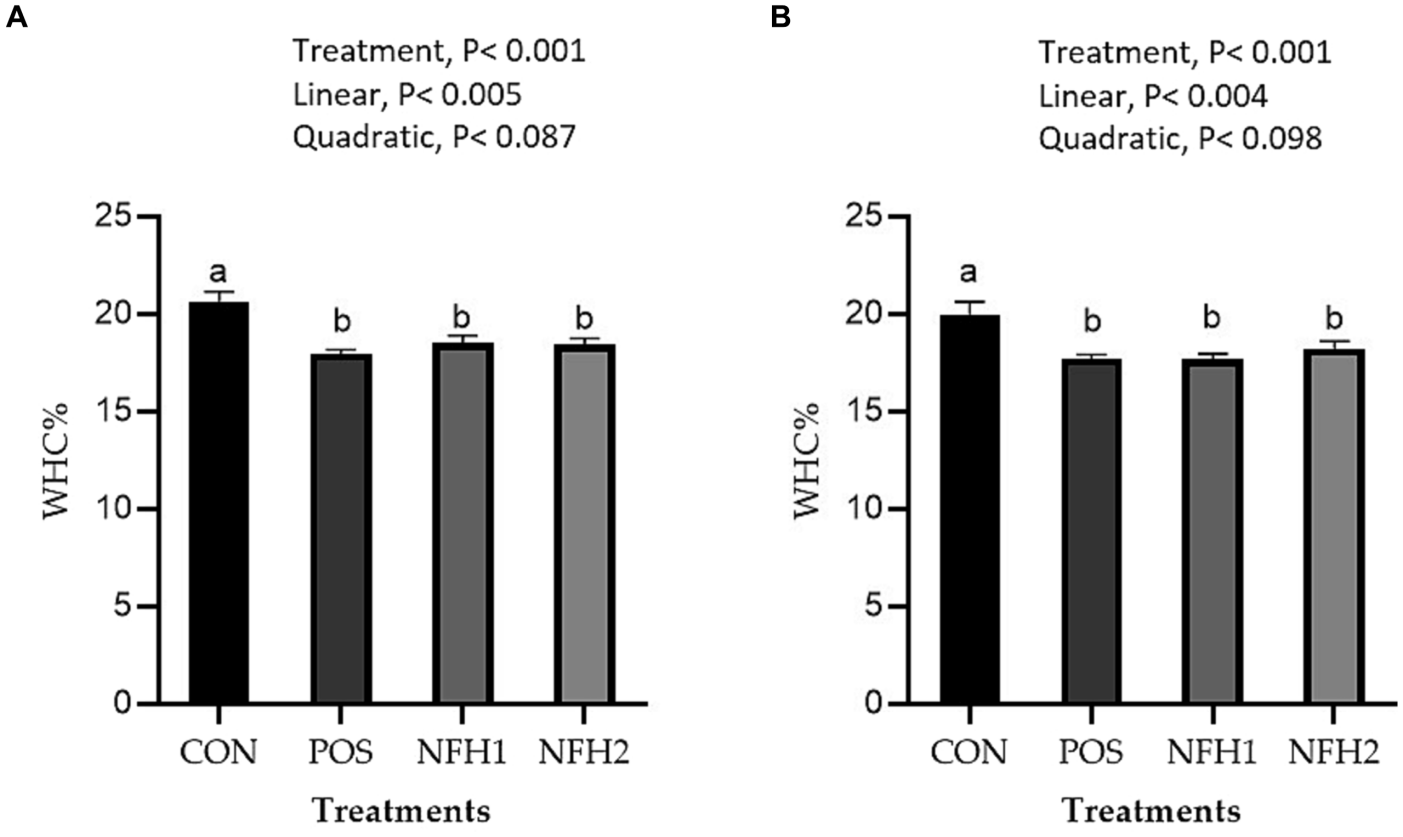
Effects of green Nano-Fe and algae on water holding capacity (WHC) of the breast **(A)** and leg **(B)** muscles in broilers at 42 days of age. Bars with different letters **(a,b)** are significantly different (*p* < 0.05). CON: negative control, POS: positive control (1 g/kg *Halimeda opuntia*), NFH1: 1 g/kg *Halimeda opuntia* with 20 mg/kg Nano-Fe, NFH2: 1 g/kg *Halimeda opuntia* with 40 mg/kg Nano-Fe, SEM: Standard error of the means (*n* = 40).

**Figure 4:**
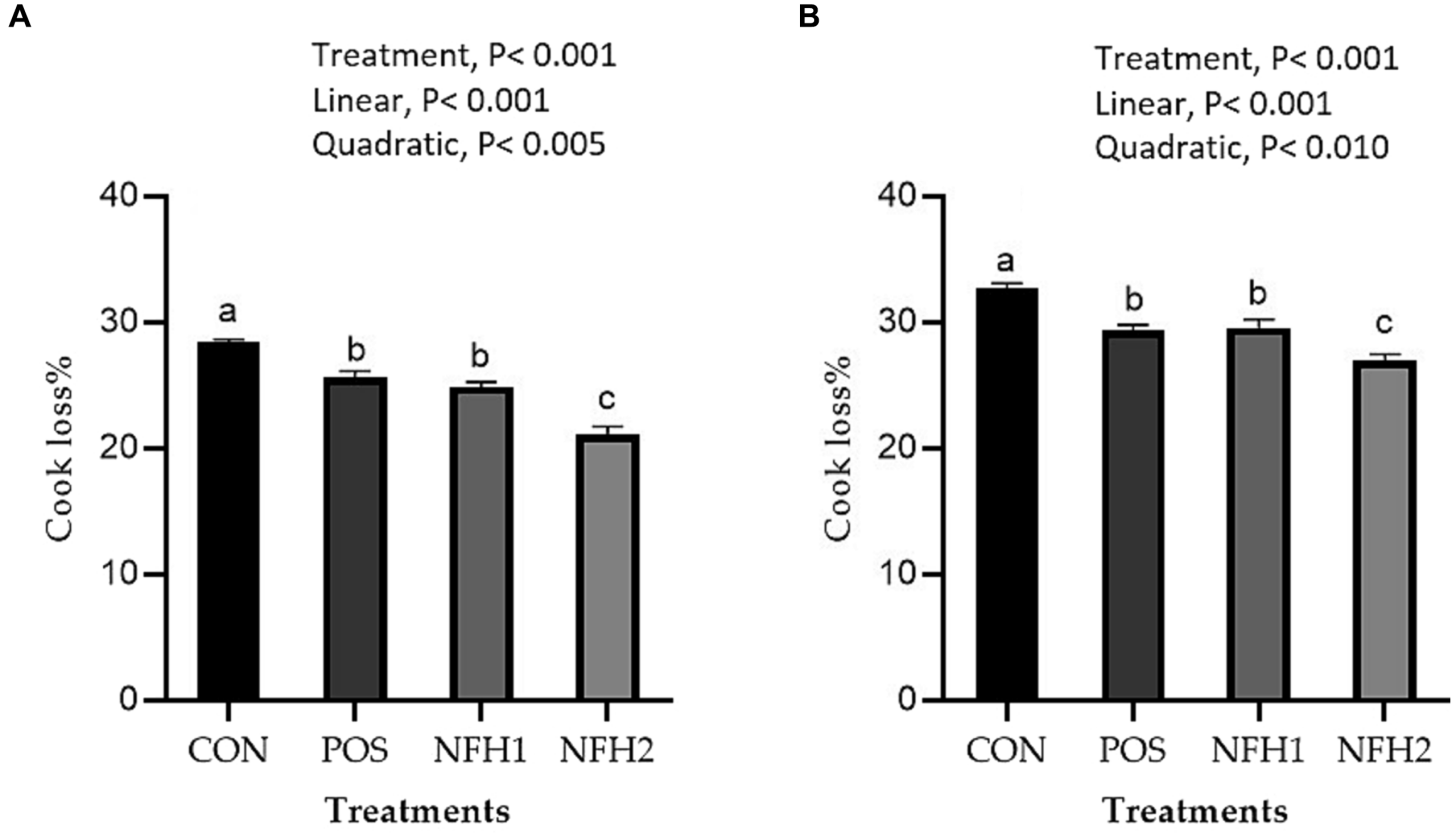
Effects of green Nano-Fe and algae on cooking loss of the breast **(A)** and leg **(B)** muscles in broilers at 42 days of age. Bars with different letters **(a–c)** are significantly different (*p* < 0.05). CON: negative control, POS: positive control (1 g/kg *Halimeda opuntia*), NFH1: 1 g/kg *Halimeda opuntia* with 20 mg/kg Nano-Fe, NFH2: 1 g/kg *Halimeda opuntia* with 40 mg/kg Nano-Fe, SEM: Standard error of the means (*n* = 40).

In terms of meat physicochemical criteria, POS and POS with Nano-Fe levels increased (*p* < 0.05) the WHC% of the breast muscles and leg muscles at 42 days of age in hot conditions (Figure 3). Supplementation of Nano-Fe to broiler diet linearly decreased WHC% in breast and leg meat compared to POS. Cook loss percentages of the breast and leg muscles at 42 days of age were reduced at POS, 20 mg/kg, and 40 mg/kg Nano-Fe levels compared to CON (Figure 4). When Nano-Fe was added to the broiler feed, cook loss in the leg and breast meat was linearly reduced in comparison to *Halimeda opuntia* (POS).

### 3.5 Iron retentions

**Figure 5:**
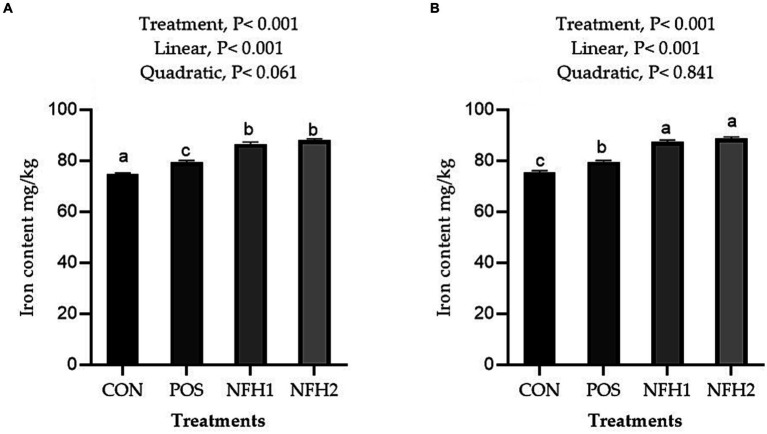
Effects of green Nano-Fe and algae on Fe content in the breast **(A)** and leg **(B)** muscles of broilers at 42 days of age. Bars with different letters **(a–c)** are significantly different (*p* < 0.05). CON: negative control, POS: positive control (1 g/kg *Halimeda opuntia*), NFH1: 1 g/kg *Halimeda opuntia* with 20 mg/kg Nano-Fe, NFH2: 1 g/kg *Halimeda opuntia* with 40 mg/kg Nano-Fe, SEM: Standard error of the means (*n* = 40).

**Figure 6:**
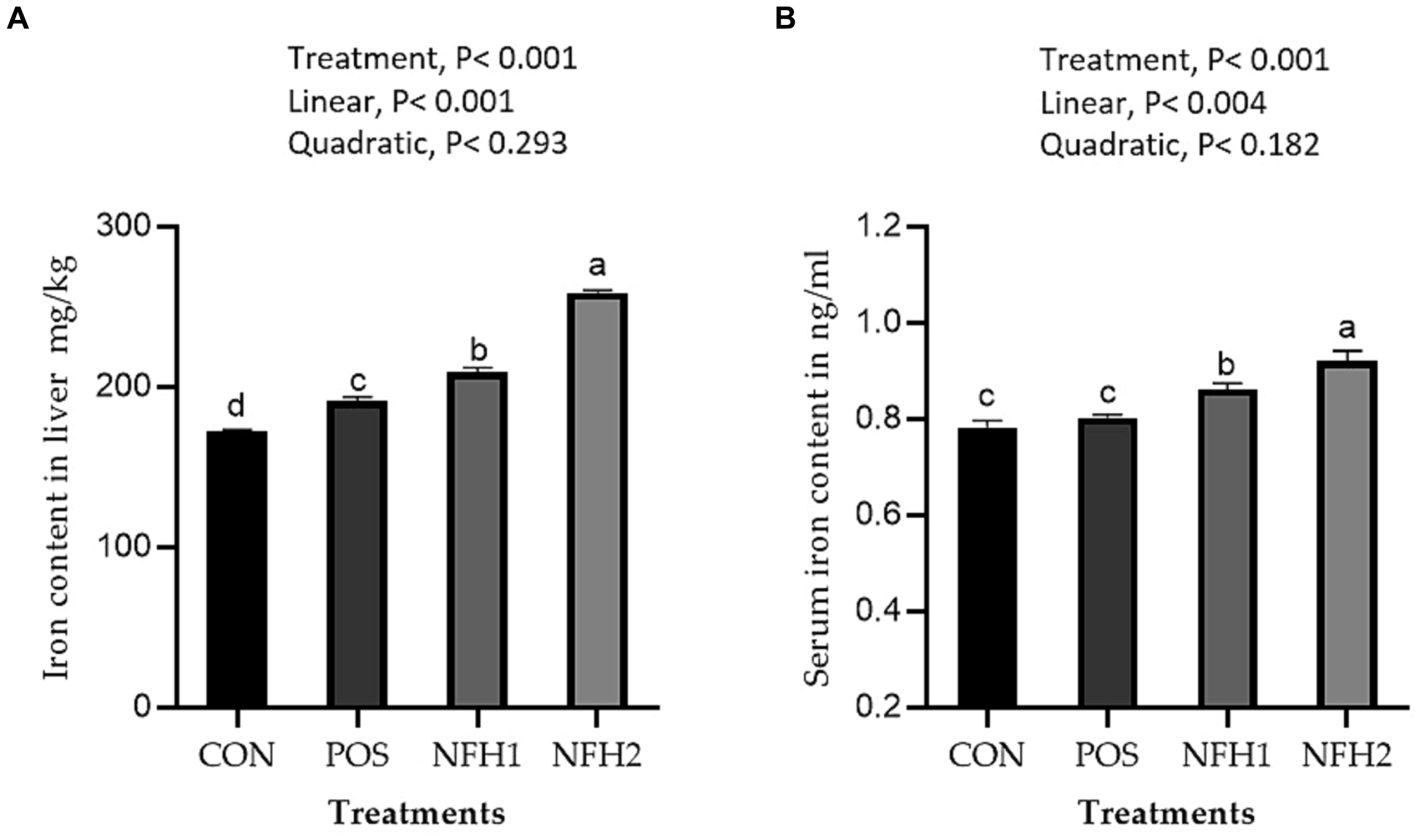
Effects of green Nano-Fe and algae on Fe content of the liver muscles **(A)** and serum **(B)** in broilers at 42 days of age. Bars with different letters **(a–c)** are significantly different (*p* < 0.05). CON: negative control, POS: positive control (1 g/kg *Halimeda opuntia*), NFH1: 1 g/kg *Halimeda opuntia* with 20 mg/kg Nano-Fe, NFH2: 1 g/kg *Halimeda opuntia* with 40 mg/kg Nano-Fe, SEM: Standard error of the means (*n* = 40).

Supplementation of algae and Nano-Fe to broiler diets improved (Linear, *p* > 0.001) the Fe contents in the breast and leg meat compared to POS at 42 days of age (Figure 5). The Fe content in the breast and leg was greater in the POS group than in the CON group. The Fe content in liver tissue was higher in the POS group compared to CON, however, there is no difference between POS and CON in Fe content in the serum of broiler chickens. The Fe content in liver tissues and serum was increased (linear, *p* < 0.05) with the increasing levels of Nano-Fe levels compared to POS (Figure 6).

## 4 Discussion

According to the current study, broiler hens fed diets containing *Halimeda opuntia* and green Nano-Fe enhanced production performance and mitigated the detrimental effects of heat stress. Heat stress is widely recognized to impair feed intake, BWG, and production performance in chickens while also increasing mortality (29–32), resulting in a loss of earnings on poultry farms. Following these negative results, meat quality (33), animal welfare (30), and immunological function (28) gradually worsen. To date, numerous mitigation methods have been introduced to lessen the detrimental effects of heat stress in poultry. Nutritional remedies have been investigated as a viable way to mitigate the negative effects of heat stress (1). Green nanotechnology feeding has the most potential as a nutritional technique, and it deserves more exploration to improve thermotolerance in chickens. Fe is an essential nutrient for chickens, and nanoparticles can improve its bioavailability. Although nanoparticles have a higher surface area, they can be more easily absorbed in the digestive tract. This increased bioavailability may contribute to improved poultry health and growth. Considering Fe is a component of hemoglobin (34) and plays a crucial role in cellular and whole-body energy and protein metabolism (35), chickens are especially vulnerable to Fe deficiency. The use of Fe nanoparticles in poultry feed has shown promise in improving feed efficiency.

In the current investigation, dietary Nano-Fe at up to 40 mg/kg increased growth performance when compared to CON. likewise, dietary treatment with POS, NFH1, and NFH2 exhibited synergistic improvements in BWG (*p* < 0.001) by 10.95, 10.30, and 14.50% compared to CON throughout the experiment period (1–42 days). Furthermore, feeding treatments with POS, NFH1, and NFH2 increased FCR (*p* < 0.006) by 8.22, 14.56, and 7.93% compared to CON, respectively. Few studies have used green Nano-Fe combined with algae as a feed additive for poultry under heat stress. Similar findings were reported by Rehman et al. (36), who found that adding xylanase and Fe oxide nanoparticles to broiler feed enhanced the FCR values at 35 days of age and raised BW by 45% compared to the CON group. The addition of 40–160 mg Fe/kg from Fe-Gly elicited higher responses; broiler chicks fed 100 mg Fe/kg exhibited the highest FCR and BWG (22). Fe oxide nanoparticles added to the diet of broiler chicks increased BW and BWG without posing any negative health risk (8). Sarlak et al. (37) observed improvements in performance indicators such as feed intake and FCR when dietary Fe was added to chicken diets compared to the CON treatment. When broiler chicks were fed Nano-Fe, their BW increased and the FCR improved (8). Compared to a CON diet without Nano-Fe, broiler diets supplemented with Nano-Fe significantly boosted BWG by 8% (38). Algae is recommended as feed additives due to their high levels of macro- and micro-elements and ability to improve the growth performance and feed efficiency of broilers (21), which is primarily due to the properties of seaweed polysaccharides that can increase the health and productivity of chickens. Furthermore, broilers’ diets containing 1 and 3.0% macroalgae *Ulva lactuca* from days 12 to 33 post-hatch revealed a significant improvement in BWG and FCR compared to CON (39). When added seaweed to broiler diets at a level of 0.5%, improved BWG, FCR, and decreased mortality rate when compared to a CON diet (40). These findings could be due to broiler immune systems and antioxidant metabolism might be strengthened by active components found in macroalgae (41), which would increase broiler productivity (42). The FCR and BW increase of broiler chicks given additional algal astaxanthin at doses of 2.3 and 4.6 mg/kg of diet showed marginal benefits (43). Additionally, broiler FCR was enhanced, and BWG was significantly boosted when 1% or 2% DHA-rich algae were added to the diet (44). The evidence of increased growth performance connected with algae-derived compounds may be inconsistent.

Furthermore, in the current study, the ammonia content of excreta was decreased with increasing green Nano-Fe levels with macroalgae compared to POS. Nano-Fe might help to upregulate the functional nitrogen metabolism pathway in intestinal bacteria, boosting the utilization of nitrogenous compounds in the host intestine and lowering ammonia elimination through excreta. Also, macroalgae may contribute to improved nutrient utilization by the broilers, leading to reduced ammonia excretion in the feces. Ammonia is produced by the microbial fermentation of uric acid and urea in feces, which causes respiratory diseases and chronic stress in livestock and poultry (45–47). Ammonia is created through the deamination of amino acids and the hydrolysis of urea. Changes in ammonia content, as well as ammonia uptake through epithelial cells, have an impact on the microbiota (48). Furthermore, as the ammonia level in the gut dropped, the compensatory effect of ammonia on intestinal cells was reduced, resulting in improvements in the intestinal barrier and histomorphology of the host intestine (49). The potential for macroalgae and microalgae to reduce fecal ammonia in broilers is an area of ongoing research and interest in the field of poultry nutrition. Ammonia is a common byproduct of the breakdown of nitrogen-containing compounds in manure, and high levels of ammonia in poultry housing can have negative effects on bird health and welfare. Seaweed contains bioactive compounds, such as certain polysaccharides and secondary metabolites, which may have the ability to influence microbial populations in the gut and reduce ammonia production (50).

In the current study, supplementations of Nano-Fe to broiler diets improved (*p* < 0.05) Fe in the breast, leg, liver, and blood. Fe is abundantly stored in the body, especially in the liver and bone marrow reticuloendothelial cells (34). Depending on the body’s Fe state, dietary demands for Fe can be regulated to enhance or decrease its rate of absorption via different recognized mechanisms (16). These mechanisms are connected to receptors on the surface of enterocytes, such as the heme carrier protein 1, which is responsible for heme-Fe absorption in the colon (51), and the divalent metal transporter 1, which may accept inorganic Fe^+2^ and immediately release it into the cytoplasm (52). Because Fe absorbs more rapidly than inorganic Fe, animal wastes including muscle tissue and blood provide greater Fe to poultry (16). Ma et al. (53) investigated the dietary Fe needs of broilers aged 1 to 21 days and discovered that 97 to 136 mg Fe/kg was necessary to sustain their complete expression in various tissues. In addition, serum ferritin levels were considerably higher in diets supplemented with 75, 150, or 300 mg/kg Fe, but not in diets supplemented with 600 mg/kg Fe (54). The Fe content of chick serum increased progressively as the Fe level in the diet increased (37). A large dose of ferrous methionine dramatically raised the hepatic Fe concentration in Ross broilers, according to research by Seo et al. (55). The Fe content of broiler liver, according to Ma (56), declined gradually when dietary Fe levels rose over 120 mg/kg. This could be because the liver was able to maintain the proper balance of Fe, preventing excessive deposition of Fe that could harm the body. The inconsistencies in the results in previous studies could be attributed to the broiler variety and their specific feed.

The effects of algae on the Fe content in broiler meat tissue are not extensively studied, and the available literature on this specific topic might be limited. However, algae, including certain types of seaweed, are known to contain various minerals, including Fe, which can be transferred to the animals consuming them (57). The bioavailability of Fe in the diet is crucial. The type of Fe present in the algae and its bioavailability could impact its transfer to the broiler meat (58). The ability of the broilers to absorb and incorporate dietary Fe into their tissues can vary based on factors such as age, health status, and genetics.

In the current investigation, supplementation of green Nano-Fe to broiler diets improved percentages of carcass dressing and reduced abdominal fat without any side effects on internal organs at 42 days of age. The results are consistent with Rehman et al. (37) showed that Fe oxide nanoparticles have a lot of potential for usage in chicken feed for large-scale meat production without any negative toxicological effects. Our results are consistent with Lin et al. (59) who found that varied amounts of Fe at 50, 70, 90, 110, 130, and 150 mg/kg did not affect the weight indices of Fabricius’ liver, kidneys, spleen, thymus, and bursa. Concerning carcass criteria, algae is recommended as feed additives due to their high levels of macro- and micro-elements and ability to improve broiler meat criteria (3). Male broilers’ diets containing 3.0% macroalgae *Ulva lactuca* revealed a significant improvement in breast muscle yield and dressing percentage compared to control (39). The improvements in carcass criterion and abdominal fat can be attributed to the favorable effects of DHA in the green alga *Ulva* on lipid utilization in serum (60). Fe, as an essential component of Fe-containing critical enzymes in broilers, plays a vital function in Fe metabolism and meat quality.

The current study found that Nano-Fe improved meat quality including WHC and cooking loss under heat stress conditions, which could be attributed to the Fe improving antioxidant activation. A recent study (61) found that Fe supplementation improved enzymatic antioxidant protection in chicken serum. While Kurtoglu et al. (62) showed that Fe-deficiency anemia lowered plasma antioxidant activities. As far as we are aware, there are no published articles on the effect of green Nano-Fe on broiler chick carcass criteria and meat quality. Fe is a required component of hemoglobin in erythrocytes and is needed by hemoglobin and myoglobin (63, 64) for oxygen delivery, storage, and usage in muscles (65). The greatest noticeable indicator of meat quality, color, is mostly determined by hemoglobin and myoglobin (66). Furthermore, Sun et al. (67) reported that adding astaxanthin-rich *Haematococcus pluvialis* to a broiler diet increased the pH of the breast muscle and lowered the WHC of the breast muscle compared to the control. The application of green Nano-Fe with algae as a carrier in broiler diets holds promise for improving production traits and meat quality as well as Fe retention. With the growing global demand for sustainable and affordable poultry nutrition alternatives, the role of green nanotechnology is gaining popularity (68–70). The use of green nanotechnology aligns with the broader goal of promoting sustainability in poultry production, optimizing resource use, minimizing environmental impact, and enhancing the overall efficiency and health of animals and poultry (71, 72). However, thorough research, including dose, safety assessments, and regulatory considerations, is necessary to ensure the responsible and effective implementation of this technology in the poultry industry.

## 5 Conclusion

Overall, the present study reveals that broiler chickens fed diets containing Nano-Fe and *Halimeda opuntia* showed synergistic enhancements growth performance, meat quality, Fe absorption, and decreased abdominal fat, but had no significant effects on internal organs. Future research ought to inquire into the impact of green Nano-Fe on immune status, microbiome, and gene expression related to immunity and heat stress.

## Data availability statement

The original contributions presented in the study are included in the article/supplementary material, further inquiries can be directed to the corresponding authors.

## Ethics statement

The animal study was approved by the Institutional Animal Care and Use Committee of the University of Alexandria, Egypt (AU08220810298). The study was conducted in accordance with the local legislation and institutional requirements.

## Author contributions

YA: Data curation, Formal analysis, Investigation, Methodology, Writing – original draft. AE: Conceptualization, Data curation, Methodology, Validation, Writing – review & editing. EE: Conceptualization, Investigation, Methodology, Writing – review & editing. AAAA-W: Data curation, Investigation, Methodology, Project administration, Writing – original draft, Writing – review & editing. JL: Validation, Writing – original draft, Writing – review & editing.
